# Rate of germline pathogenic sequence variants in cancer susceptibility genes in an Israeli pediatric and adolescent cancer cohort: a single institute experience

**DOI:** 10.1007/s10689-025-00489-1

**Published:** 2025-08-08

**Authors:** Dana Nahom, Zehavit Frenkel, Amos Toren, Eitan Friedman, Iris Kventsel

**Affiliations:** 1 Faculty of Medicine, Tel Aviv University, Tel Aviv, Israel; 2 Sheba Medical Center, Ramat Gan, Israel; 3 The Meirav High Risk Clinic, Sheba Medical Center, Tel-Hashomer, Israel; 4 Center for Preventive Personalized Medicine, Assuta Medical Center, Tel Aviv, Israel; 5 Pediatric Hematology Oncology Division, Sheba Medical Center, Ramat Gan, Israel

**Keywords:** Inherited predisposition to cancer, Childhood and adolescent cancer, Multi-cancer predisposition gene panel testing (MCPGT), Pathogenic sequence variants (PSVs), Cancer predisposing gene (CPG)

## Abstract

Multi-cancer predisposition gene panel testing (MCPGT) enables simultaneous deep-coverage genotyping of multiple CPGs (cancer predisposing genes) and detects germline pathogenic sequence variants (PSVs). Reported PSV carrier rates among pediatric and adolescent cancer patients range from 8 to 17.6%, with variability attributed to ethnic background, the number of genes tested, cancer phenotypes, and patient selection criteria. This study aimed to assess the rate and spectrum of germline PSVs in consecutive pediatric and adolescent cancer patients treated at the Sheba Medical Center, a tertiary medical center. All cancer patients aged 0–18 years treated between 01.2021 and 12.2022 were offered MCPGT. Overall, 257 eligible cancer patients were treated during the study period, of whom 116 Israeli patients underwent MCGPT (Invitae, San Francisco, CA), with complete data available for 108. The range of malignancies included central nervous system (CNS) tumors (n = 45), solid tumors (n = 37), hematological malignancies (n = 14), and retinoblastoma (RB) (n = 12). PSVs were detected in 17/108—15.7% of patients, with the highest rates in patients with RB (7/12, 58.3%) and CNS tumors (6/45, 13.3%). A significant association was found between younger age at diagnosis and PSV carrier status (4.7 years in carriers vs. 9.3 years in non-carriers, *p* = 0.003). Fulfilling Jongmans' criteria was correlated with PSV detection. The study highlights the importance of genetic testing in children meeting specific clinical criteria, particularly those diagnosed with RB and CNS tumors. Further studies with larger, ethnically diverse cohorts are needed to validate these findings to expand the scientific basis for personalized care strategies.

**Supplementary Information:**

The online version contains supplementary material available at 10.1007/s10689-025-00489-1.

## Introduction

Inherited predisposition to cancer is estimated to contribute to ~ 5–10% of all adult cancer cases [1]. While the assignment of inherited predisposition to cancer in childhood is primarily clinical (based on well-accepted criteria [2]), the presence of a *bona fide* pathogenic germline sequence variant (PSV) in a major cancer susceptibility gene indicates the carrier individual’s lifetime risk of developing cancer that is substantially higher than the general population or individual’s lifetime risk of developing cancer, unlike the risk definition determined by multiple Single Nucleotide Polymorphisms (SNPs) in a polygenic risk score (PRS). For example, germline PSVs in the *BRCA1* and *BRCA2* genes are associated with a significantly increased risk of breast and ovarian cancers in women, and breast and prostate cancers in men [3, 4]. Since environmental exposure to carcinogens accumulates over time, cancers in adults are more likely to develop as a result of environmental exposure, whereas in pediatric cancer patients, inherited genetic factors presumably play a more significant role in carcinogenesis [5, 6].

Currently, the criteria recommended by international societies and clinically adopted define who should be offered genetic counselling and testing from among cancer patients diagnosed at 0–18 years. A commonly used panel of criteria, Jongmans’ criteria [2], includes: (1) Family history of cancer, including: two or more malignancies diagnosed in childhood (≤ 18 years); a first-degree relative (parent or sibling) with cancer before the age of 45; two or more second-degree relatives with cancer diagnosed before age 45 on the same side of the family; or consanguinity between the parents of the affected child. (2) Diagnosis of a tumor associated with an increased likelihood of an underlying cancer predisposition syndrome (see Supplementary Table 1). Also included are cancers typically seen in adults (e.g., colorectal, ovarian, and basal cell carcinoma) diagnosed in a child. (3) The presence of two malignancies in a child, provided that the second malignancy is not clearly a treatment-related neoplasm expected for the given therapy. (4) Childhood cancer associated with congenital anomalies or specific phenotypic features, such as: Congenital anomalies (affecting organs, bones, orofacial clefts, eyes, ears, urogenital tract, etc.), Facial dysmorphisms, Intellectual disability, Abnormal growth (e.g., head circumference, birth weight, asymmetric growth), Skin abnormalities (e.g., > 2 café-au-lait macules, vascular anomalies, multiple benign tumors of the skin), Hematologic disorders (e.g., anemia, thrombocytopenia, neutropenia, pancytopenia), Immune deficiency. (5) Excessive treatment-related toxicity beyond what is expected for age, diagnosis, or protocol. Fulfilling only one of these criteria is deemed sufficient for offering genetic analysis of pediatric cancer cases.

There is an ongoing debate in the medical community about the best approach to genetic testing of pediatric cancer patients: perform universal screening by offering genetic testing for all known cancer predisposition genes to every patient diagnosed with cancer up to age 18 years or refer for genetic counselling and testing only those patients who fulfil well-established criteria. This debate involves also cost-effectiveness analyses [7], the preferred genotyping technique—performing MCPGT or conducting a targeted test focused only on genes suspected based on the patient’s phenotype [8]. The rate of inherited cancer predisposition among pediatric patients as determined by detecting PSVs in phenotypically relevant genes varies between 8 and 17.6% [7, 9, 10] depending on cancer type, diagnostic method (e.g., targeted, predefined gene panels or comprehensive whole-exome sequencing), and whether genetic testing was conducted based on clinical suspicion of an inherited predisposition or offered non-selectively to all cancer patients.

This study evaluated the prevalence of PSVs carriers in genes known to be associated with a hereditary predisposition to cancer among consecutive Israeli oncology patients aged 0–18 years who were treated in the Pediatric Hemato-oncology Department at Sheba Medical Center between 2021 and 2022.

## Materials and methods

### Study population

All Israeli patients (Jewish and non-Jewish) aged 0–18 years diagnosed with cancer and treated at the Pediatric Hemato-Oncology department at the Sheba Medical Center, Tel-Hashomer, Israel between January 2021 to December 2022, without applying any additional selection criteria, were eligible and offered participation in the study. Non-Israeli patients were not included in the study because genetic testing for them was based on clinical criteria rather than universally due to insurance issues. In addition, individuals who underwent previous genetic testing for PSVs in a Cancer Susceptibility Gene (CSG) were not eligible for participation. The patient population at Sheba Medical Center includes individuals from a wide geographical area across the central, most populated part of Israel. Additionally, some patients treated at the Sheba Medical Center reside in Northern and Southern Israel and were referred for treatment at Sheba as it is a tertiary medical center.

### Collection of clinical and demographic data

Medical and demographic information on the study population were collected from the medical records as they appear in the “Chameleon” information system of the Sheba Medical Center. The collected data included sex, year of birth, ethnic origin, parental consanguinity, age at symptom onset, tumor type and anatomical location, tumor stage and grade and family history of malignancies. The fulfillment of the Jongmans’ criteria [2] was assessed retrospectively through a review of medical records by the team of researchers. In our study, components 1–3 of the Jongmans’ criteria were applied, whereas criteria 4 and 5 were not applied due to lack of available phenotypic and toxicity data in the cohort.

### Genetic analysis

DNA was extracted from peripheral blood leukocytes or saliva collected mostly before treatment initiation (except in cases of accessibility issues at that time). For patients with active leukemia, the samples were collected during remission. Samples were analyzed by the Multi-Cancer, Pediatric Solid Tumors, Pediatric Hematologic Malignancies, Pediatric Nervous System/Brain Tumors multigene panel of Invitae (https://www.invitae.com/—San Francisco, CA, USA—Supplementary Table 2). The pathogenicity assignment of the detected sequence variants was provided by the Invitae pipeline, as previously described [11]. To emphasize, no further filtering steps taken by an independent laboratory to re-assign pathogenicity to the variants beyond the Invitae assignment pipeline. In the current study, we focused on P/LPSVs in CSG that are either relevant to the patient phenotype (as primary findings) or on PSVs in CSG that are irrelevant to the phenotype but still affect CSG (secondary findings).

### Statistical analysis

Statistical analyses were performed using the Mann–Whitney **U** test for non-normally distributed quantitative variables. For qualitative data, the Chi-Squared Test was used. The Fisher’s Exact Test was employed when small sample sizes were involved. All statistical analyses were conducted using SPSS Statistics 21 software, with a *p* value < 0.05 considered statistically significant.

### Ethics statement

The study was approved by the Sheba Institutional Review Board (ethics) committee (approval number: 0184-23-SMC; date of approval: April 30, 2023). For all pediatric participants, clinical informed consent was obtained from their parents or legal guardians before the genetic testing, as is standard practice in clinical testing. The parents or legal guardians signed a clinical informed consent form before the genetic testing, as is standard practice in clinical tests. Thus, they signed consent forms for the test to be performed clinically, with the test results disclosed in the context of formal genetic counseling. Ethical approval was obtained for the analysis of all results in a de-identified manner, without any direct contact with the patients; thus, specific informed consent for the analysis reported herein was waived by the Sheba IRB.

## Results

Participant characteristics—Overall, 140/257 (54.5%) eligible patients whose parents or legal guardians provided consent to the proposed clinical genetic testing. Reasons for declining to participate were not recorded. Moreover, some participating physicians did not consistently offer genetic testing to their patients, for reasons that were not recorded. Of consenting patients, 23 non-Israeli citizens and one patient who was diagnosed after age 18 years were excluded; eight additional participants were excluded due to technical issues, of whom four dues to the cessation of Invitae’s activities in Israel, resulting in a final analyzable sample of 108 patients. Table 1 displays the spectrum of tumors, patients’ sex and age groups at cancer diagnosis in all 108 patients. Of participants, 61/108 were male (56.5%) and 47/108—female (43.5%), mean age at diagnosis was 8.6 ± 6.2 years (SD), with a median of 8.5 years (range 0–18 years). Ethnic origin was available for 85 patients, with the majority being of Ashkenazi Jewish (n = 31) or of mixed Ashkenazi/non-Ashkenazi origin (n = 24). Of the data available for non-participants, there were significant differences between participants and non-participants in tumor types (Supplementary Table 3).

**Table 1:** The distribution of tumor types by age and sex categories

Disease	Age at diagnosis				Sex		Subtotal
	0–1 year	1–5 years	5–10 years	10–18 years	Female	Male	
Retinoblastoma	7	5	0	0	4	8	12
Ewing sarcoma	0	1	1	4	2	4	6
Central nervous system*	7	11	11	16	19	26	45
Osteosarcoma	0	1	0	6	5	2	7
Rhabdomyosarcoma	1	0	2	1	3	1	4
Hematological malignancy**	0	4	1	9	4	10	14
Other solid tumors***	2	5	4	9	10	10	20
Subtotal	17	27	19	45	47	61	108

*Detailed in Supplementary Table 4

**Detailed in Supplementary Table 5

***Detailed in Supplementary Table 6

Genetic analysis—Overall, 293 sequence variants were identified across the 308 genes analyzed in all 108 genotyped patients. Notably, no sequence variants were detected in 20 patients. Of these variants, 48 (16.4%) were classified as pathogenic, 6 (2.1%) as likely pathogenic, 206 (70.3%) as variants of uncertain significance (VUS), 7 (2.4%) as likely benign, and 26 (8.8%) as benign. Thus, a total of 54 P/LPSVs were identified. Of these, 8 variants were classified as secondary findings and are listed in Table 4, 17 variants were associated with a patient-relevant phenotype and are presented in Table 2, and 29 additional P/LP variants that were not associated with a phenotype are listed in Supplementary Table 8.

**Table 2:** Germline pathogenic/ likely pathogenic sequence variants and the associated phenotype

Diagnosis	Age at diagnosis	Gene	Variant	Protein	P/LPSVs^a^	Effect on transcript/protein	Meets Jongmans’ criteria	Family history of cancer^b^	Genotype
Breast cancer	16 years	*TP53*	c.394A > G	p. Lys132Glu	P	Missense	Yes (P2)	No	Heterozygous
Myelodysplasia	1 month	*FANCC*	c.456 + 4A > T (Intronic)	–	P	Splice Site	Yes (F2)	Yes	Homozygous
Low-grade glioma	17 years	*NF1*	c.1173_1174insGT	p. Gln392Valfs*21	P	Frameshift	Yes (F1)	Yes	Heterozygous
Low-grade glioma	9 years	*PTPN11*	c.794g > A	p. ARG265Gln	P	Missense	Yes (F3)	Yes	Heterozygous
Optic glioma	3 years	*NF1*	c.1721 + 3A > G (Intronic)	–	P	Splice Site	Yes (P1, F2)	Yes	Heterozygous
Low-grade glioma	3 years	*NBN*	c.1903A > T	p. Lys635*	P	Nonsense	No	No	Heterozygous
Medulloblastoma	12 years	*TP53*	c.695T > C	p. Ile232Thr	P	Missense	Yes (P1)	No	Heterozygous
Medulloblastoma	2 years	*SUFU*	c.37_53del	p. Thr13Trpfs*29	P	Frameshift	Yes (P1)	No	Heterozygous
Neuroblastoma	2 months	*BLM*	c.98 + 1G > T (Splice donor)	–	LP	Splice Site	No	No	Heterozygous
Retinoblastoma	1 month	*RB1*	Deletion (Exons 3–27)	–	P	Deletion (In Frame Deletion)	Yes (P1)	No	Heterozygous
Retinoblastoma	2 months	*RB1*	c.751C > T	*p.Arg251	P	Nonsense	Yes (P1)	No	Heterozygous
Retinoblastoma	3 months	*RB1*	c.1005del	p. Leu335Phefs*14	P	Frameshift	Yes (P1)	Yes	Heterozygous
Retinoblastoma	6 months	*RB1*	c.763C > T	p. Arg255*	P	Nonsense	Yes (P1, F2)	Yes	Heterozygous
Retinoblastoma	9 months	*NF1*	c.3661C > G	p. (Leu1221Val)	LP	Missense	Yes (P1)	No	Heterozygous
Retinoblastoma	1.5 years	*RB1*	Deletion (Exon 2)	–	P	Deletion (In Frame Deletion)	Yes (P1)	No	Heterozygous
Retinoblastoma	2 years	*RB1*	c.1072C > T	p. (Arg358*)	P	Nonsense	Yes (P1)	No	Possibly mosaic
Sarcoma of the liver	3 years	*TP53*	c.818 G > A	p. Arg273His	P	Missense	Yes (F1, P2)	Yes	Heterozygous

One or more first-degree relatives with cancer under the age of 45

More than two second-degree relatives with cancer under the age of 45 on the same side of the family, Parents of a child with malignancy who are related by blood

Jongmans’ criteria—abbreviations used in table

Familial criteria (F):

F1—Two or more malignancies in the family diagnosed under the age of 18

F2—At least one first-degree relative with cancer diagnosed under the age of 45

F3—Two or more second-degree relatives with cancer diagnosed under the age of 45 on the same side of the family

F4—Parents of a child with malignancy who are consanguineous (biologically related)

Personal Criteria (P):

P1—Specific childhood tumor types known to be associated with cancer predisposition syndromes (see Supplementary Table 1)

P2—Tumor types typically seen in adults but occurring in childhood (e.g., colorectal cancer, ovarian cancer, pheochromocytoma)

P3—Presence of two primary malignancies in the patient, with at least one diagnosed before age 18

P4—Malignancy occurring as part of a syndrome that also includes congenital anomalies (e.g., Gorlin syndrome, Beckwith–Wiedemann syndrome)

^a^Pathogenic/likely pathogenic sequence variants

^b^Family history—Two or more malignancies in the family under the age of 18

Overall, 17/108 (15.7%) study participants harbored P/LPSVs in phenotypically relevant genes: 9/17 males (52.9%) and 8/17 females (47.1%). After excluding RB patients from the sample, the rate of PSV carriers was 10/96 (10.4%). PSVs were most frequently detected in patients diagnosed with RB (7/12—58.3%) and CNS tumors (6/45—13.3%).

The *Rb1* gene was the most commonly mutated gene, with P/LPSVs were noted in 6/12 (50%) RB patients; P/LPSVs were noted in the *NF1* gene in 3 patients: 1/12 (8.3%) patients with RB and 2/3 (66.6%) patients with low-grade glioma. In *TP53*, P/LPSVs were identified in three patients who were diagnosed with breast cancer, liver sarcoma, and medulloblastoma. P/LPSVs and associated phenotypes are shown in Table 2.

Mean age at diagnosis among patients harboring P/LPSVs was 4.7 ± 5.9 years, with a median age of 2 years. For patients with no detected P/LPSVs, the mean age was 9.3 ± 6.05 years, with a median age of 9 years (*p* = 0.003). The main contributors to the age differences between carriers and non-carriers were RB-diagnosed patients. After excluding RB patients from the age-centered analysis, mean age for P/LPSVs carriers was 6.2 ± 7.6 years, with a median age of 6 years, and 9.7 ± 5.9 years, with a median age of 9.5 years for non-carriers (*p* = 0.271).

Of 17 P/LPSVs carriers, 15 patients (88.2%) met one or more of Jongmans’ criteria [2], whereas 30/81 (37%) non-carriers of any P/LPSVs met one or more of Jongmans’ criteria (Supplementary Table 7). For 10 patients (10.9%), no information regarding family cancer history was available in the medical records, and therefore, it could not be determined whether they met these criteria. The detection rate of a PSV among those who met Jongmans’ criteria was 33.3% (15/45), compared with 3.8% (2/53) among patients who do not meet any of these criteria, a statistically significant difference (*p* = 0.00012). The two patients who did not meet the Jongmans’ criteria carried monoallelic PSVs in *BLM* and *NBN*, genes associated with autosomal recessive cancer predisposition syndromes, and a single heterozygous variant is generally insufficient to confer a clinically meaningful cancer risk. Therefore, these patients did not meet the Jongmans’ criteria, and their exclusion from further genetic evaluation in accordance with these guidelines seems justifiedt.

Seven patients were found to carry more than one PSV in the CPS panel (Table 3).

**Table 3:** Combinations of variants and diagnoses in patients found to carry PSVs in two or more genes

#	Diagnosis	Gene 1	Gene 2	Gene 3
1	Retinoblastoma	*Rb1*	*RBM8A*	–
2	Retinoblastoma	*ATM*	*Rb1*	*ABCG8*
3	Low-grade glioma	*NBN*	*RECQL4*	–
4	Optic glioma	*NF1*	*BRCA2*	–
5	Retinoblastoma	*Rb1*	*CHEK2*	–
6	Neuroblastoma	*BLM*	*MUTYH*	–
7	Low-grade glioma	*PTPN11*	*TNFRSF13B*	–

### Secondary findings

In 8 patients, secondary findings (as defined by the ACMG [12]) were noted (Table 4). These include P/LPSVs in genes that predispose individuals to the development of adult cancers. However, the significance and clinical implication of early detection recommendations in children are not sufficiently clear. Additionally, PSVs were detected in several genes included in the genotyping panel, which are rarely associated with genetic predisposition to childhood malignancies or did not match the patient’s phenotype; therefore, they were not defined as positive results and did not require reporting according to ACMG guidelines (see Supplementary Table 8).

**Table 4:** Information on the various variants found in genes classified as secondary findings

#	Diagnosis	Gene	Variant	P/LP^a^ Increased Risk	Genotype
1	Glioblastoma multiforme	*BRCA1*	Deletion (Exons 1–2)	P	Heterozygous
2	Glioblastoma multiforme		c.68_69del (p. Glu23Valfs*17)	P	Heterozygous
3	Optic glioma	*BRCA2*	c.5946del (p. Ser1982Argfs*22)	P	Heterozygous
4	Diffuse meningal melanocytosis	APC * (X2)	c.3920T > A (p. Ile1307Lys)	Increased risk allele	Heterozygous
5	Low-grade glioma				
6	Glioblastoma multiforme	*MSH2*	c.970_971del (p. Gln324Valfs*8)	P	Heterozygous
7	Neuroblastoma	MUTYH* (X2)	c.536A > G (p.Tyr179Cys)	P	Heterozygous
8	Low-grade glioma				

*The variant was found in two different patients

^a^Pathogenic/ likely pathogenic sequence variants

## Discussion

In the current study, 17/108 (15.7%) Israeli pediatric and adolescent cancer patients, whose parents or legal guardians provided consent and were treated at a single, tertiary referral medical center, were found to carry P/LPSVs in clinically and phenotypically relevant cancer susceptibility genes. The carrier rate in this study was in the same range as previously reported in different populations worldwide, who were similarly selected for genotyping (ranging from 8.5 to 15.3%) (Table 5).

**Table 5:** PSVs detection rates in the current study and in previously published studies

Study	Origin	Screening method	Percentage of patients with P/LPSVs^a^
Current Study	Israel	Universal	15.74% (17/108)
Gargallo et al. [13]	Spain	Criteria-based	15.3% (26/170)
Byrialsen et al. [14]	Denmark	Universal	10.6% (21/198)
Sylvester et al. [15]	Australia	Criteria-based	11.8% (9/76)
Zhang et al. [10]	USA	Universal	8.5% (95/1120)
Bakhuizen et al. [8]	Netherlands	Universal	9% (94/1052)
van Tilburg et al. [16]	Germany	Universal	7.5% (39/519)

^a^Pathogenic/likely pathogenic sequence variants

Some differences between study populations may account for the wide range of detection rates. Importantly, the specific cancer types included in the analysis seem to be the key factor underlying these differences. For example, in the current study, there were more RB cases and fewer hematological malignancy patients compared to the study by Gargallo et al. [13]. In the current study, most RB patients harbored a PSV in the *Rb1* gene. Moreover, after excluding patients with RB from the analysis, the carrier rate was 10.4% (10/96), similar to the rates reported in other studies genotyping consecutive age-matched cancer cases.

Notably, in the study by van Tilburg et al. [16], the percentage of PSVs was lower than that reported in other studies that used similar methodology. This difference may be attributed to the inclusion of patients diagnosed between the ages of 18–21 in that study. As in our study, patients with hematologic malignancies were underrepresented relative to their prevalence in the general pediatric cancer population (8.7%). Additionally in the study by Byrjalsen et al. [14], patients with previously known CPSs prior to malignancy onset, such as Down syndrome and neurofibromatosis type 1 (NF1), were included. In contrast, the studies by Bakhuizen et al. [8], Zhang et al. [10], Sylvester et al. [15] and Gargallo et al. [13] included only newly diagnosed CPSs and excluded patients with previously known CPSs, similar to the current study. These differences in the inclusion criteria may underlie the differences in the rate of detection of PSV’s between Byrjalsen et al. [14] and Zhang et al. [10]. Bakhuizen et al. [8] reported that the overall prevalence of PSVs detected by universal screening was 9%, although it should be noted that this included pathogenic variants not necessarily associated with the patient phenotype.

In the current study, the PSV carrier rate among patients who met Jongmans’ Criteria [2] was significantly higher than that among those who did not meet these criteria (33. 3% (15/45) vs. 3.8% (2/53), *p* = 0.00012). Of the 17 PSV carriers, 15 (88.2%) fulfilled one or more of the Jongmans’ criteria. These results are in line with those of previous studies. Byrjalsen et al. [14] reported that among cases who met either the Jongmans’ and/or MIPOGG [7] criteria, carrier rate of P/LPSVs was 81%. Gargallo et al. [13] reported that 15/16 (94%) patients identified as carriers of P/LPSVs met the Jongmans’ criteria. Similarly, in the study by Bakhuizen et al. [8] 26/250 (10%) patients fulfilling the MIPOGG [7] criteria were PSV carriers, whereas only 1/483 (0.2%) of those not fulfilling these criteria were found to be PSV carrier. Thus, it seems that applying these clinical criteria may increase the yield of detecting PSVs in cancer cases in the pediatric age group. However, the number of patients who do not fulfill Jongmans’ criteria found to harbor a pathogenic variant in a CPG (3.8%—2/53) may be considered as an indication to support the more permissive approach of universal genetic screening of pediatric cancer patients. Clearly, more data need to be accumulated before such a decision can be made for more pediatric cases from diverse populations.

The genes most commonly harboring P/LPSVs in the current study were *Rb1*, *NF1*, and *TP53* (Table 2). These findings are consistent with those of several previous studies reporting genotyping of non-Israeli cohorts [8, 10, 13, 14]. One issue that possibly affects these rates is whether “positive findings” include PSVs in genes that are clinically irrelevant to paediatric patients such as *BRCA1/BRCA2* and to what extent were PSV in genes that are unrelated to the phenotype reported back to the cases or are considered as “secondary findings”.

In the current study, 13.3% (6/45) of patients with CNS tumors carried P/LPSVs. This finding is consistent with a recent study [17] demonstrating the high prevalence of genetic predisposition to cancer among children with brain tumors—15–21% of CNS tumors are associated with CPS. That study also noted that SHH-medulloblastomas were enriched for CPS, with current data suggesting a prevalence of up to 40%. These results reinforce the understanding that a significant genetic component contributes to the development of these tumors, and highlight the importance of comprehensive genetic testing as part of the clinical evaluation of children with CNS tumors. In line with the study by Nakano et al. [18] who reported that low-grade gliomas (LGG) are associated with neurofibromatosis type 1 (NF1), *SUFU* PSVs were detected in patients with medulloblastoma and Gorlin syndrome, and that *TP53* PSVs—in patients with medulloblastoma—our cohort showed similar associations between these phenotypes and these mutated genes (Table 6).

**Table 6:** Malignancies that are part of specific hereditary cancer syndromes

Tumor type	PSVs^a^	Hereditary cancer syndromes
Medulloblastoma	*SUFU*	Gorlin syndrome
Low-grade glioma	*NF1*	Neurofibromatosis type 1
Low-grade glioma	*NF1*	Neurofibromatosis type 1
Hepatic sarcoma	*TP53*	Li–Fraumeni syndrome (met classic criteria)
Breast carcinoma	*TP53*	Li–Fraumeni syndrome (met Chompret criteria [19])
Medulloblastoma	*TP53*	Li–Fraumeni syndrome (met Chompret criteria)

^a^Pathogenic/likely pathogenic sequence variants

In 8/108 (7.4%) patients reported herein, PSVs were detected in “actionable” genes. However, these genes are irrelevant to the genotyped individual, as the associated cancer type is either unrelated to the current phenotype or clinically non-actionable because of the age of the genotyped individual. For example, PSVs in the *BRCA1* and *BRCA2* genes are associated with a significantly increased lifetime risk of developing breast and ovarian cancer, and in Israel, testing is offered only from the age of 25, when an early detection scheme in carrier women is being offered (https://www.nccn.org/guidelines/category_2). Moreover, the *APC* risk allele (p. I1307K) is not recommended for testing before the age of 40, if at all. The decision to report the presence of adult-onset cancer-related genes when genotyping pediatric cancer cases is a debated topic, while most guidelines emphasize that reporting should be focused on data that are imminently relevant to the affected child’s health, avoiding any possible ethical infringement on child autonomy to decide as an adult on being informed of these test results and potential detrimental psychological effects and family ties, the benefits to healthy adult family members to detect a PSV in CSG that may alter their surveillance recommendation needs to be taken into consideration [20].

Zhang et al. [10] reported that germline PVs in *TP53* are present in 4.5% (50/1120) of children and adolescents with cancer. Luo et al.[21] reported that germline PVs in *TP53* are present in 23/2788 (0.8%) of children and adolescents with cancer after somatic TP53 analysis. In our study, we identified germline TP53 PVs in 2.7% (3/108) of patients a rate that is within the range previously reported.

The rate of secondary findings in our study (8/108, 7.4%) was higher than that in previous studies, which ranged between 1.2 and 1.7% [9, 22]. The differences in rates may be attributed to the fact that the literature primarily focuses on MMR and *BRCA1/2* genes, whereas our study included additional genes. Additionally, it is known that the frequency of founder *BRCA1/BRCA2* PSVs among Ashkenazi Jews is higher than that reported in other populations worldwide [23], which may explain the relatively high rate of secondary findings in the current study. Another plausible explanation is that previous studies focusing on pediatric cancer patients have reported all PSV in all CSG as “primary findings” regardless of the relevance of these PSV to the specific patients’ phenotype and the well accepted cancer risks associated with a PSV in these specific genes. In the study by McGee et al. [24]. Secondary findings were identified in 3% (33/1018) of the genotyped patients, which is similar to the rate found in our study. The McGee et al. [24] study genotyped a larger number of genes associated with adult-onset cancer, including *APC*, *ATM, AXIN2, BRCA1, BRCA2, BRIP1, CDH1, CHEK2, EPCAM, FLCN, GREM1, MET, MLH1, MSH2, MSH3, MSH6, MUTYH, NTHL1, PALB2, PMS2, POLD1, POLE, RAD51C,* and *RAD51D.*

Another study supporting our findings relating to co-carriership of two PSVs in CSGs was published by Laitman et al. [25], who reported that dual carriage of PSV in major cancer-associated genes is indeed rare, but is associated with more complex phenotypes and an increased risk of developing multiple types of cancer at a younger age.

Our study found that a younger age at diagnosis was associated with an increased risk of carrying germline PSVs, and patients diagnosed at an average age of 4.7 years had a higher likelihood of being carriers than those diagnosed at an older average age (*p* = 0.003). However, after excluding patients with RB from the sample, the difference in age at diagnosis between carriers and non-carriers was no longer statistically significant. Thus, for non-RB pediatric cancer patients, age at diagnosis may not be a good distinctive predictive indication for finding a PSV in a CSG.

The current study has several limitations. This study included only 108 genotyped children (from potentially eligible cohort of 257 patients). Genetic testing was performed in 140/257 Israeli age eligible patients (54.5%) based on the treating physician’s decision and the family’s consent. Such selection bias leads to a sample of patients who do not fully represent a universal screening approach. Specifically, there is an underrepresentation of patients with hematologic malignancies compared to their actual prevalence in the general pediatric and adolescent cancer population, and an overrepresentation of patients with RB. Notably, of 300–400 new cancer cases diagnosed annually in Israel in the 0–18 age group, 40–55% are hematological malignancies (leukemia and lymphoma combined), 15–20% CNS tumors, and 5–10% neuroblastoma (Supplementary Table 9). Additional limitations include the limited range of tumors, the incompleteness of data (e.g., in 10 patients no data on family history; in 23, no data on ethnicity) and that all patients were treated at a single medical center in Israel. Combined these caveats limit the generalizability of the findings to the overall population of children with malignancies in Israel and elsewhere.

In conclusion, this study suggests a preliminary and tentative association between a specific clinical and genetic profile and the rate of detection of P/LPSVs in phenotypically relevant CSG in pediatric cancer patients, especially in patients diagnosed with RB and CNS tumors and those with other tumor types who fulfil the Jongmans’ criteria. Further studies with larger sample sizes are needed to strengthen this association and to aid in developing personalized protocols for genetic analysis of patients and their families.

## Supplementary Information

Below is the link to the electronic supplementary material.

Supplementary Material 1

## Data Availability

No datasets were generated or analysed during the current study.

## References

[1] Rossini L, Durante C, Bresolin S, Opocher E, Marzollo A, Biffi A (2022) Diagnostic strategies and algorithms for investigating cancer predisposition syndromes in children presenting with malignancy. Cancers 14(15):3741. 10.3390/cancers14153741 35954404 10.3390/cancers14153741 PMC9367486

[2] Jongmans MCJ, Loeffen JLCM, Waanders E, Hoogerbrugge PM, Ligtenberg MJL, Kuiper RP et al (2016) Recognition of genetic predisposition in pediatric cancer patients: an easy-to-use selection tool. Eur J Med Genet 59(3):116–125 26825391 10.1016/j.ejmg.2016.01.008

[3] Van Der Kolk DM, De Bock GH, Leegte BK, Schaapveld M, Mourits MJE, De Vries J et al (2010) Penetrance of breast cancer, ovarian cancer and contralateral breast cancer in BRCA1 and BRCA2 families: high cancer incidence at older age. Breast Cancer Res Treat 124(3):643–651 20204502 10.1007/s10549-010-0805-3

[4] Pritchard CC, Mateo J, Walsh MF, De Sarkar N, Abida W, Beltran H et al (2016) Inherited DNA-repair gene mutations in men with metastatic prostate cancer. N Engl J Med 375(5):443–453 27433846 10.1056/NEJMoa1603144 PMC4986616

[5] Kratz CP, Jongmans MC, Cavé H, Wimmer K, Behjati S, Guerrini-Rousseau L et al (2021) Predisposition to cancer in children and adolescents. Lancet Child Adolesc Heal 5(2):142–154. 10.1016/S2352-4642(20)30275-3 10.1016/S2352-4642(20)30275-3 33484663

[6] Kratz CP (2024) Re-envisioning genetic predisposition to childhood and adolescent cancers. Nat Rev Cancer 25(February):109–128. 10.1038/s41568-024-00775-7 39627375 10.1038/s41568-024-00775-7

[7] Goudie C, Witkowski L, Cullinan N, Reichman L, Schiller I, Tachdjian M et al (2021) Performance of the McGill interactive pediatric oncogenetic guidelines for identifying cancer predisposition syndromes. JAMA Oncol 7(12):1806–1814. 10.1001/jamaoncol.2021.4536 34617981 10.1001/jamaoncol.2021.4536 PMC8498936

[8] Bakhuizen JJ, van Dijk F, Koudijs MJ, Bladergroen RS, Bon SBB, Hopman SMJ, Kester LA, Kranendonk MEG, Loeffen JLC, Smetsers SE, Sonneveld E, Tachdjian M, de Vos-Kerkhof E, Cather MCJ (2024) A comparison of clinical selection-based genetic testing with extensive phenotype-agnostic germline sequencing to diagnose genetic predisposition in children with cancer: a prospective cohort. Lancet Child Adolesc Health 8(10):751–761. 10.1016/S2352-4642(24)00144-5 39159644 10.1016/S2352-4642(24)00144-5

[9] Kim J, Gianferante M, Karyadi DM, Hartley SW, Frone MN, Luo W et al (2021) Frequency of pathogenic germline variants in cancer-susceptibility genes in the childhood cancer survivor study. JNCI Cancer Spectr. 5(2):pkab007. 10.1093/jncics/pkab007 34308104 10.1093/jncics/pkab007 PMC8023430

[10] Zhang J, Walsh MF, Wu G, Edmonson MN, Gruber TA, Easton J et al (2015) Germline mutations in predisposition genes in pediatric cancer. N Engl J Med 373(24):2336–2346. 10.1056/NEJMoa1508054 26580448 10.1056/NEJMoa1508054 PMC4734119

[11] Nykamp K, Anderson M, Powers M, Garcia J, Herrera B, Ho YY et al (2017) Sherloc: a comprehensive refinement of the ACMG-AMP variant classification criteria. Genet Med 19(10):1105–1117. 10.1038/gim.2017.37 28492532 10.1038/gim.2017.37 PMC5632818

[12] Miller DT, Lee K, Abul-Husn NS, Amendola LM, Brothers K, Chung WK et al (2023) ACMG SF v3.2 list for reporting of secondary findings in clinical exome and genome sequencing: a policy statement of the American College of Medical Genetics and Genomics (ACMG). Genet Med 25(8):100866 37347242 10.1016/j.gim.2023.100866 PMC10524344

[13] Gargallo P, Oltra S, Yáñez Y, Juan-Ribelles A, Calabria I, Segura V et al (2021) Germline predisposition to pediatric cancer, from next generation sequencing to medical care. Cancers (Basel) 13(21):5339. 10.3390/cancers13215339 34771502 10.3390/cancers13215339 PMC8582391

[14] Byrjalsen A, Hansen TVO, Stoltze UK, Mehrjouy MM, Barnkob NM, Hjalgrim LL et al (2020) Nationwide germline whole genome sequencing of 198 consecutive pediatric cancer patients reveals a high frequency of cancer prone syndromes. PLoS Genet 16(12):1–24. 10.1371/journal.pgen.1009231 10.1371/journal.pgen.1009231 PMC7787686 33332384

[15] Sylvester DE, Chen Y, Grima N, Saletta F, Padhye B, Bennetts B et al (2022) Rare germline variants in childhood cancer patients suspected of genetic predisposition to cancer. Genes Chromosom Cancer 61(2):81–93. 10.1002/gcc.23006 34687117 10.1002/gcc.23006

[16] Van Tilburg CM, Pfaff E, Pajtler KW, Langenberg KPS, Fiesel P, Jones BC et al (2021) The pediatric precision oncology inform registry: clinical outcome and benefit for patients with very high-evidence targets. Cancer Discov 11(11):2764–2779. 10.1158/2159-8290.CD-21-0094 34373263 10.1158/2159-8290.CD-21-0094 PMC9414287

[17] Hansford JR, Das A, McGee RB, Nakano Y, Brzezinski J, Scollon SR et al (2024) Update on cancer predisposition syndromes and surveillance guidelines for childhood brain tumors. Clin Cancer Res 30(11):2342–2350. 10.1158/1078-0432.CCR-23-4033 38573059 10.1158/1078-0432.CCR-23-4033 PMC11147702

[18] Nakano Y, Rabinowicz R, Malkin D (2023) Genetic predisposition to cancers in children and adolescents. Curr Opin Pediatr 35(1):55–62. 10.1097/MOP.0000000000001197 36354292 10.1097/MOP.0000000000001197

[19] Bougeard G, Renaux-Petel M, Flaman JM, Charbonnier C, Fermey P, Belotti M et al (2015) Revisiting Li–Fraumeni syndrome from TP53 mutation carriers. J Clin Oncol 33(21):2345–2352. 10.1200/JCO.2014.59.5728 26014290 10.1200/JCO.2014.59.5728

[20] Kesserwan C, Friedman Ross L, Bradbury AR, Nichols KE (2016) The advantages and challenges of testing children for heritable predisposition to cancer. Am Soc Clin Oncol Educ Book 36:251–269. 10.1200/EDBK_160621 10.1200/EDBK_160621 27249705

[21] Luo M, Wong D, Zelley K, Wu J, Schubert J, Denenberg EH et al (2024) Identification of TP53 germline variants in pediatric patients undergoing tumor testing: strategy and prevalence. J Natl Cancer Inst 116(8):1356–1365. 10.1093/jnci/djae102 38702830 10.1093/jnci/djae102

[22] Li S, Nguyen-Dumont T, Southey MC, Hopper JL (2023) Re: heterozygous *BRCA1* and *BRCA2* and mismatch repair gene pathogenic variants in children and adolescents with cancer. J Natl Cancer Inst 115(6):757–759. 10.1093/jnci/djad056 37004193 10.1093/jnci/djad056 PMC10248828

[23] Roa BB, Boyd AA, Volcik K, Richards CS (1996) Ashkenazi Jewish population frequencies for common mutations in BRCA1 and BRCA2. Nat Genet 14(2):185–187 8841191 10.1038/ng1096-185

[24] McGee RB, Oak N, Harrison L, Xu K, Nuccio R, Blake AK et al (2023) Pathogenic variants in adult-onset cancer predisposition genes in pediatric cancer: prevalence and impact on tumor molecular features and clinical management. Clin Cancer Res 29(7):1245–1251. 10.1158/1078-0432.CCR-22-2482 10.1158/1078-0432.CCR-22-2482 PMC10642481 36693186

[25] Laitman Y, Niskakoski A, Bernstein-Molho R, Koskinen L, Rabina D, Koskenvuo J et al (2024) Phenotypes of carriers of two mutated alleles in major cancer susceptibility genes. Breast Cancer Res Treat 208(3):589–595. 10.1007/s10549-024-07454-z 39102164 10.1007/s10549-024-07454-z PMC11522137

