# Supplementary material for: Rate of germline pathogenic sequence variants in cancer susceptibility genes in an Israeli pediatric and adolescent cancer cohort: a single institute experience
Source: Fam Cancer. 2025 Aug 8;24(3):62. doi: 10.1007/s10689-025-00489-1 (PMC12334452; doi:10.1007/s10689-025-00489-1)
Supplement: Supplementary file 1 — Supplementary Material 1 [file 10689_2025_489_MOESM1_ESM.pdf]

Supplementary material

Supplementary Table 1- Specific and rare childhood tumors for which genetic testing is recommended for all affected individuals.

Adrenocortical carcinoma	Endolymphatic sac tumors	Low hypodiploid ALL	Optic glioma	Pineoblastoma
Atypical teratoid - rhabdoid tumor	Hemangioblastoma	Malignant peripheral nerve sheath tumor	Ovarian steroli-leydig cell tumor	Retinoblastoma
Cerebellar gangliocytoma	Hepatoblastoma	Medullary thyroid carcinoma	Pleuropulmonary blastoma	Schwannoma
Choroid plexus carcinoma	Juvenile Myelomonocytic Leukemia	Medulloblastoma	Pituitary blastoma	Subependymal giant cell tumor

Supplementary Table 2- List of 308 Genes Analyzed in the Panel.

#	GENE	TRANSCRIPT
1	*ABCB7*	NM_004299.4
2	*ABCG5*	NM_022436.2
3	*ABCG8*	NM_022437.2
4	*ABRAXAS1*	NM_139076.2
5	*ACD*	NM_001082486.1
6	*ACTN1*	NM_001130004.1
7	*ADA*	NM_000022.2
8	*ADA2*	NM_001282225.1
9	*ADAMTS13*	NM_139025.4
10	*AIP*	NM_003977.3
11	*AK1*	NM_000476.2
12	*AK2**	NM_001625.3
13	*AKT1*	NM_005163.2
14	*ALAS2*	NM_000032.4
15	*ALDOA*	NM_000034.3
16	*ALK*	NM_004304.4
17	*ANK1*	NM_000037.3
18	*ANKRD26**	NM_014915.2

19	*ANO6**	NM_001025356.2
20	*AP2S1*	NM_004069.4
21	*AP3B1*	NM_003664.4
22	*APC**	NM_000038.5
23	*ARPC1B*	NM_005720.3
24	*ATM**	NM_000051.3
25	*ATR**	NM_001184.3
26	*AXIN2*	NM_004655.3
27	*BAP1*	NM_004656.3
28	*BARD1*	NM_000465.3
29	*BCOR*	NM_017745.5
30	*BLM*	NM_000057.3
31	*BLOC1S3*	NM_212550.4
32	*BLOC1S6*	NM_012388.3
33	*BMPR1A*	NM_004329.2
34	*BPGM*	NM_199186.2
35	*BRCA1*	NM_007294.3
36	*BRCA2*	NM_000059.3
37	*BRIP1*	NM_032043.2
38	*BUB1B*	NM_001211.5
39	*C15orf41*	NM_001130010.2
40	*CARD11*	NM_032415.5
41	*CARMIL2*	NM_001013838.1
42	*CASP8*	NM_001228.4
43	*CASR*	NM_000388.3
44	*CBL*	NM_005188.3
45	*CD19*	NM_001770.5
46	*CD27*	NM_001242.4
47	*CD40*	NM_001250.5
48	*CD40LG*	NM_000074.2
49	*CD81*	NM_004356.3

50	*CDAN1*	NM_138477.2
51	*CDC42*	NM_001791.3
52	*CDC73*	NM_024529.4
53	*CDH1*	NM_004360.3
54	*CDK4*	NM_000075.3
55	*CDKN1B*	NM_004064.4
56	*CDKN1C*	NM_000076.2
57	*CDKN2A (p14ARF)*	NM_058195.3
58	*CDKN2A (p16INK4a)*	NM_000077.4
59	*CEBPA*	NM_004364.4
60	*CEBPE*	NM_001805.3
61	*CEP57**	NM_014679.4
62	*CFTR**	NM_000492.3
63	*CHEK2*	NM_007194.3
64	*CLPB*	NM_030813.5
65	*COL4A1*	NM_001845.5
66	*CPA1*	NM_001868.3
67	*CSF3R*	NM_000760.3
68	*CTC1*	NM_025099.5
69	*CTLA4*	NM_005214.4
70	*CTNNA1*	NM_001903.3
71	*CTPS1*	NM_001905.3
72	*CTR9*	NM_014633.4
73	*CTRC*	NM_007272.2
74	*CXCR4*	NM_003467.2
75	*CYCS*	NM_018947.5
76	*DDX41*	NM_016222.3
77	*DIAPH1*	NM_005219.4
78	*DICER1**	NM_177438.2
79	*DIS3L2**	NM_152383.4
80	*DKC1*	NM_001363.4

81	*DNAJC21*	NM_001012339.2
82	*DOCK8*	NM_203447.3
83	*DTNBP1*	NM_032122.4
84	*EFL1**	NM_024580.5
85	*EGFR*	NM_005228.3
86	*EGLN1**	NM_022051.2
87	*ELANE*	NM_001972.2
88	*ENG**	NM_000118.3
89	*EPAS1**	NM_001430.4
90	*EPB41*	NM_004437.3; NM_001166005.1
91	*EPB42*	NM_000119.2
92	*EPCAM**	NM_002354.2
93	*EPOR*	NM_000121.3
94	*ERBB2**	NM_004448.3
95	*ERCC4*	NM_005236.2
96	*ERCC6L2*	NM_020207.4
97	*ETV6*	NM_001987.4
98	*EXT1*	NM_000127.2
99	*EXT2*	NM_207122.1
100	*EZH2**	NM_004456.4
101	*FADD*	NM_003824.3
102	*FANCA*	NM_000135.2
103	*FANCB*	NM_001018113.1
104	*FANCC*	NM_000136.2
105	*FANCD2**	NM_033084.3
106	*FANCE*	NM_021922.2
107	*FANCF*	NM_022725.3
108	*FANCG*	NM_004629.1
109	*FANCI*	NM_001113378.1
110	*FANCL**	NM_018062.3
111	*FANCM*	NM_020937.2

112	*FAS*	NM_000043.5
113	*FASLG*	NM_000639.2
114	*FCHO1*	NM_001161357.1
115	*FERMT3*	NM_031471.5
116	*FH**	NM_000143.3
117	*FLCN*	NM_144997.5
118	*FLI1*	NM_002017.4
119	*G6PC*	NM_000151.3
120	*G6PC3*	NM_138387.3
121	*G6PD*	NM_001042351.2
122	*GALNT12*	NM_024642.4
123	*GATA1*	NM_002049.3
124	*GATA2*	NM_032638.4
125	*GCLC*	NM_001498.3
126	*GEN1*	NM_182625.3
127	*GFI1**	NM_005263.3
128	*GNA11*	NM_002067.4
129	*GP1BA**	NM_000173.6
130	*GP6*	NM_001083899.2
131	*GP9*	NM_000174.4
132	*GPC3**	NM_004484.3
133	*GPI*	NM_000175.3
134	*GREM1**	NM_013372.6
135	*GSR**	NM_000637.3
136	*GSS*	NM_000178.2
137	*GYPC**	NM_002101.4
138	*HAX1*	NM_006118.3
139	*HK1*	NM_000188.2
140	*HOXB13*	NM_006361.5
141	*HPS1*	NM_000195.4
142	*HPS3*	NM_032383.4

143	*HPS4*	NM_022081.5
144	*HPS5*	NM_181507.1
145	*HPS6*	NM_024747.5
146	*HRAS*	NM_005343.2
147	*HTRA2*	NM_013247.4
148	*IKZF1*	NM_006060.6
149	*IL2RA*	NM_000417.2
150	*IL2RB*	NM_000878.3
151	*ITGA2B*	NM_000419.3
152	*ITGB3*	NM_000212.2
153	*ITK*	NM_005546.3
154	*JAGN1*	NM_032492.3
155	*JAK2*	NM_004972.3
156	*KCNN4*	NM_002250.2
157	*KIF1B**	NM_015074.3
158	*KIF23*	NM_138555.3
159	*KIT*	NM_000222.2
160	*KLF1*	NM_006563.4
161	*KRAS*	NM_004985.4
162	*LPIN2*	NM_014646.2
163	*LYST*	NM_000081.3
164	*LZTR1*	NM_006767.3
165	*MAGT1*	NM_032121.5
166	*MAX**	NM_002382.4
167	*MC1R*	NM_002386.3
168	*MCM4*	NM_005914.3
169	*MDM2**	NM_002392.5
170	*MECOM*	NM_001105078.3
171	*MEN1**	NM_130799.2
172	*MET**	NM_001127500.1
173	*MITF*	NM_000248.3

174	*MLH1**	NM_000249.3
175	*MLH3**	NM_001040108.1
176	*MPL*	NM_005373.2
177	*MRE11*	NM_005591.3
178	*MSH2**	NM_000251.2
179	*MSH3**	NM_002439.4
180	*MSH6**	NM_000179.2
181	*MUTYH*	NM_001128425.1
182	*MYH9*	NM_002473.5
183	*MYSM1*	NM_001085487.2
184	*NBN*	NM_002485.4
185	*NF1**	NM_000267.3
186	*NF2*	NM_000268.3
187	*NHP2*	NM_017838.3
188	*NOP10*	NM_018648.3
189	*NT5C3A*	NM_016489.12
190	*NTHL1*	NM_002528.6
191	*P2RY12*	NM_022788.4
192	*PALB2*	NM_024675.3
193	*PALLD*	NM_001166110.1
194	*PARN*	NM_002582.3
195	*PDGFRA*	NM_006206.4
196	*PFKM*	NM_000289.5
197	*PGK1*	NM_000291.3
198	*PHOX2B**	NM_003924.3
199	*PIEZO1**	NM_001142864.3
200	*PIK3CA*	NM_006218.2
201	*PIK3CD*	NM_005026.3
202	*PIK3R1*	NM_181523.2
203	*PKLR*	NM_000298.5
204	*PLA2G4A*	NM_024420.2

205	*PMS2**	NM_000535.5
206	*POLD1**	NM_002691.3
207	*POLE*	NM_006231.3
208	*POT1*	NM_015450.2
209	*PRKAR1A*	NM_002734.4
210	*PRKCD*	NM_006254.3
211	*PRSS1**	NM_002769.4
212	*PTCH1*	NM_000264.3
213	*PTCH2*	NM_003738.4
214	*PTEN**	NM_000314.4
215	*PTPN11*	NM_002834.3
216	*RAB27A*	NM_004580.4
217	*RAC2*	NM_002872.4
218	*RAD50*	NM_005732.3
219	*RAD51C*	NM_058216.2
220	*RAD51D*	NM_002878.3
221	*RASGRP1*	NM_005739.3
222	*RASGRP2*	NM_153819.1
223	*RB1**	NM_000321.2
224	*RBM8A*	NM_005105.4
225	*RECQL**	NM_002907.3
226	*RECQL4**	NM_004260.3
227	*REST*	NM_005612.4
228	*RET*	NM_020975.4
229	*RHAG*	NM_000324.2
230	*RHOH*	NM_004310.4
231	*RINT1*	NM_021930.4
232	*RMRP*	NR_003051.3
233	*RNF43*	NM_017763.5
234	*RPL11*	NM_000975.3
235	*RPL15*	NM_002948.3

236	*RPL19**	NM_000981.3
237	*RPL26*	NM_000987.3
238	*RPL35A*	NM_000996.2
239	*RPL5*	NM_000969.3
240	*RPS10*	NM_001014.4
241	*RPS19*	NM_001022.3
242	*RPS20*	NM_001023.3
243	*RPS24*	NM_033022.3
244	*RPS26*	NM_001029.3
245	*RPS29*	NM_001032.4
246	*RPS7*	NM_001011.3
247	*RTEL1*	NM_001283009.1
248	*RUNX1*	NM_001754.4
249	*SAMD9*	NM_017654.3
250	*SAMD9L*	NM_152703.4
251	*SDHA**	NM_004168.3
252	*SDHAF2*	NM_017841.2
253	*SDHB*	NM_003000.2
254	*SDHC**	NM_003001.3
255	*SDHD*	NM_003002.3
256	*SEC23B*	NM_006363.4
257	*SH2D1A*	NM_002351.4
258	*SLC2A1*	NM_006516.2
259	*SLC37A4*	NM_001164277.1
260	*SLC4A1*	NM_000342.3
261	*SLX4*	NM_032444.2
262	*SMAD4*	NM_005359.5
263	*SMARCA4*	NM_001128849.1
264	*SMARCB1*	NM_003073.3
265	*SMARCD2*	NM_001098426.1
266	*SMARCE1*	NM_003079.4

267	*SPINK1*	NM_003122.4
268	*SPRED1*	NM_152594.2
269	*SPTA1*	NM_003126.2
270	*SPTB*	NM_000347.5
271	*SRP54*	NM_003136.3
272	*SRP72*	NM_006947.3
273	*STAT3*	NM_139276.2
274	*STIM1*	NM_003156.3
275	*STK11*	NM_000455.4
276	*STK4*	NM_006282.3
277	*STN1*	NM_024928.4
278	*STXBP2*	NM_006949.3
279	*SUFU*	NM_016169.3
280	*TAZ*	NM_000116.4
281	*TBXA2R*	NM_001060.5
282	*TCN2*	NM_000355.3
283	*TERC*	NR_001566.1
284	*TERT*	NM_198253.2
285	*THPO*	NM_000460.3
286	*TIMM50*	NM_001001563.3
287	*TINF2*	NM_001099274.1
288	*TMEM127*	NM_017849.3
289	*TNFRSF13B*	NM_012452.2
290	*TP53*	NM_000546.5
291	*TPI1*	NM_000365.5
292	*TPP2*	NM_003291.2
293	*TSC1**	NM_000368.4
294	*TSC2*	NM_000548.3
295	*TUBB1*	NM_030773.3
296	*UBE2T*	NM_014176.3
297	*USB1*	NM_024598.3

298	*VHL*	NM_000551.3
299	*VIPAS39*	NM_022067.3
300	*VPS13B*	NM_017890.4
301	*VPS33B*	NM_018668.4
302	*VPS45*	NM_007259.4
303	*WAS*	NM_000377.2
304	*WIPF1*	NM_001077269.1
305	*WRAP53*	NM_018081.2
306	*WRN**	NM_000553.4
307	*WT1*	NM_024426.4
308	*XIAP*	NM_001167.3

An asterisk (*) next to a gene indicates a limitation (e.g., sequencing of only part of the exon rather than the entire exon).

Supplementary Table 3- Comparison of cancer types between the group of patients genotyped and those that were not (p <0.0001).

**Tumor Type**	**Referred n, (%)**	**Not Referred n, (%)**
**Hematologic malignancies**	19 (23.2%)	63 (76.8%)
**Central nervous system tumors**	55 (80.9%)	13 (19.1%)
**Solid tumors**	51 (56 %)	40 (44%)
**Retinoblastoma**	15 (93.8%)	1 (6.2%)
**Total**	140 (54.5%)	117 (45.5%)

*** Supplementary Table 4- Age distribution at diagnosis of different CNS tumors identified in the study, along with a detailed breakdown of CNS tumor types.**

**Central Nervous System Tumor Types**	**Age at diagnosis**				**Subtotal**
	**0-1 y**	**1-5 y**	**5-10 y**	**10-18 y**	
**Medulloblastoma**	0	2	0	2	4
**Low-grade glioma**	3	5	5	6	19
**Glioblastoma Multiforme**	1	1	2	3	7
**Optic Glioma**	1	1	0	1	3
**Diffuse Meningeal Melanocytosis**	0	1	0	0	1
**Germinoma**	0	0	0	2	2
**Anaplastic Astrocytoma**	0	0	1	0	1
**Ganglioglioma + Pleomorphic Xanthoastrocytoma**	0	0	1	0	1
**Pinealoblastoma**	1	0	0	0	1
**Diffuse Leptomeningeal Melanomatosis**	0	0	1	0	1
**Diffuse Leptomeningial Glioneuronal**	0	0	0	1	1
**Ependymoma**	0	1	0	0	1
**Subependymoma**	0	0	0	1	1
**Spinal Schwannoma**	0	0	1	0	1
**Ganglioma**	1	0	0	0	1
**Subtotal**	7	11	11	16	**45**

Supplementary Table 5- Age distribution at diagnosis of various hematologic malignancies and a detailed breakdown of malignancy types in the study. **

Hematological malignancy	Age at diagnosis				Subtotal
	0-1 y	1-5 y	5-10 y	10-18 y	
Acute Lymphoblastic Leukemia (ALL)	0	3	0	1	4
Anaplastic Large Cell Lymphoma	0	0	1	0	1
Marginal Zone Lymphoma	0	0	0	1	1
Acute Myeloid Leukemia (AML)	0	0	0	3	3
Hodgkin Lymphoma	0	0	0	3	3
T cell Lymphoblastic Lymphoma	0	0	0	1	1
Myelodysplasia	0	1	0	0	1
Subtotal	0	4	1	9	**14**

***** Supplementary Table 6- Age distribution at diagnosis of various solid tumors and a detailed breakdown of tumor types in the study.**

Other solid tumors	Age at diagnosis				Subtotal
	**0-1 y**	**1-5 y**	**5-10 y**	**10-18 y**	
**Ganglioneuroblastoma**	0	2	0	0	2
**Synovial Sarcoma**	0	0	0	1	1
**Desmoplastic Fibroma**	0	0	0	1	1
**Neuroblastoma**	1	0	0	0	1
**Immature Ovarian Teratoma**	0	1	0	1	2
**Breast Carcinoma**	0	0	0	1	1
**Juvenile granulosa cell tumor**	0	0	1	0	1
**Hepatic Sarcoma**	0	1	0	0	1
**Squamous Cell Carcinoma of the Mandible**	0	0	0	1	1
**Hepatoblastoma**	0	1	0	0	1
**Lipoblastoma**	1	0	0	0	1
**Chondrosarcoma**	0	0	1	0	1
**Wilms' Tumor**	0	0	1	0	1
**Peritoneal Mesothelioma**	0	0	1	0	1
**Adenocarcinoma of the Colon**	0	0	0	1	1
**Immature Teratoma**	0	0	0	3	3
**Subtotal**	2	5	4	9	**20**

Supplementary Table 7- Jongmans Criteria Fulfillment for Patients Without a P/LPSVs

#	Diagnosis	Age at diagnosis	Meets Jongmans' Criteria	Family History of Cancer
1	Adenocarcinoma of the Colon	17	Yes (P2)	Yes
2	ALL	2	Yes (F2)	Yes
3	AML	18	No	No
4	AML	14	No	No
5	AML	13	No	No
6	Anaplastic astrocytoma	9	No	No
7	Anaplastic large cell lymphoma	7	No	No
8	B-ALL	3	No	No
9	BCP-ALL	16	Yes (F2)	Yes
10	BCP-ALL	5	Yes (F2)	Yes
11	Chondrosarcoma	9	No	No
12	Desmoplastic fibroma	16	No	No
13	Diffuese leptomeningeal melanomatosis	8	Yes (F3)	No
14	Diffuse leptomeningial glioneuronal	14	No	No
15	Diffuse meningal melanocytosis	2	No	No
16	Ependymoma	5	No	No
17	Ewing sarcoma	18	No	No
18	Ewing sarcoma	18	Not available (N/A)	Not available (N/A)
19	Ewing sarcoma	8	Not available (N/A)	Not available (N/A)
20	Ewing sarcoma	12	No	No
21	Ewing sarcoma	3	No	No
22	Ewing sarcoma	14	Not available (N/A)	Not available (N/A)
23	Ganglioglioma+Pleomorphic Xanthoastrocytoma	9	No	No
24	Ganglioma	1	Yes (F3)	Yes
25	Ganglioneuroblastoma	4	Yes (F2)	No
26	Ganglioneuroblastoma	2	No	No
27	Germinoma	16	No	No
28	Glioblastoma multiforme	18	No	No
29	Glioblastoma multiforme	17	Yes (F2)	Yes
30	Glioblastoma multiforme	0	No	No
31	Glioblastoma multiforme	9	Yes (F3)	No
32	Glioblastoma multiforme	2	No	No
33	Glioblastoma multiforme	16	No	No
34	Glioblastoma Multiforme	8	No	No
35	Low-grade glioma	13	No	No
36	Low-grade glioma	4	No	No
37	Low-grade glioma	5	No	No
38	Low-grade glioma	9	No	No
39	Low-grade glioma	0	No	No
40	Low-grade glioma	18	No	No

41	Low-grade glioma	0	No	No
42	Low-grade glioma	15	No	No
43	Low-grade glioma	4	No	No
44	Low-grade glioma	17	No	No
45	Glioma of brainstem	7	No	No
46	Hepatoblastoma	3	Yes (P1)	No
47	Hodgkin lymphoma	16	No	No
48	Hodgkin lymphoma	14	Yes (F2)	Yes
49	Hodgkin lymphoma	18	Not available (N/A)	Not available (N/A)
50	Immature teratoma	12	No	No
51	Juvenile granulosa cell tumor	6	No	No
52	Lipoblastoma	1	No	No
53	Lymphoma of the Lacrimal Sac	11	No	No
54	Medulloblastoma	16	Yes (P1, F3)	Yes
55	Medulloblastoma	2	Yes (P1)	No
56	Mixed cell non germinoma	16	Yes (F2)	No
57	Optic glioma	1	Yes (P1)	No
58	Optic glioma	3	Yes (P1, F2)	Yes
59	Osteosarcoma	17	Not available (N/A)	Not available (N/A)
60	Osteosarcoma	4	Not available (N/A)	Not available (N/A)
61	Osteosarcoma	14	No	No
62	Osteosarcoma	16	No	No
63	Osteosarcoma	14	No	No
64	Osteosarcoma	17	Not available (N/A)	Not available (N/A)
65	Osteosarcoma	13	Yes (F2)	Yes
66	Ovarian Mature Cystic Teratoma	4	Yes (F3)	No
67	Ovarian teratoma	11	No	No
68	Ovary Immature Teratoma	18	Not available (N/A)	Not available (N/A)
69	Ovary Immature Teratoma	13	No	No
70	Peritoneal Mesothelioma	8	No	No
71	Low-grade glioma	1	No	No
72	Low-grade glioma	5	No	No
73	Low-grade glioma	14	Yes (F2)	Yes
74	Low-grade glioma	9	Yes (F3)	No
75	Low-grade glioma	6	Yes (P4, F3)	Yes
76	Pinealoblastoma	0	Yes (P1)	No
77	Retinoblastoma	2	Yes (P1, F3)	No
78	Retinoblastoma	2	Yes (P1)	Yes
79	Retinoblastoma	3	Yes (P1)	No
80	Retinoblastoma	4	Yes (P1)	No
81	Retinoblastoma	0	Yes (P1, F3)	No
82	Rhabdomyosarcoma	6	Yes (F3)	Yes
83	Rhabdomyosarcoma	1	No	No

84	Rhabdomyosarcoma	10	No	No
85	Rhabdomyosarcoma	18	Not available (N/A)	Not available (N/A)
86	Spinal Schwannoma	10	Yes (P1)	No
87	Squamouns cell carcinoma	17	No	No
88	Subependymoma	13	No	No
89	Synovial sarcoma	13	Yes (F2)	Yes
90	T cell lymphoblastic lymphoma	15	Not available (N/A)	Not available (N/A)
91	Wilms' Tumor	6	No	No

Jongmans' Criteria – Abbreviations Used in Table

Familial Criteria (F):

F1 – Two or more malignancies in the family diagnosed under the age of 18

F2 – At least one first-degree relative with cancer diagnosed under the age of 45

F3 – Two or more second-degree relatives with cancer diagnosed under the age of 45 on the same side of the family

F4 – Parents of a child with malignancy who are consanguineous (biologically related)

Personal Criteria (P):

P1 – Specific childhood tumor types known to be associated with cancer predisposition syndromes (see Supplementary Table 9)

P2 – Tumor types typically seen in adults but occurring in childhood (e.g., colorectal cancer, ovarian cancer, pheochromocytoma)

P3 – Presence of two primary malignancies in the patient, with at least one diagnosed before age 18

P4 – Malignancy occurring as part of a syndrome that also includes congenital anomalies (e.g., Gorlin syndrome, Beckwith-Wiedemann syndrome)

Supplementary Table 8- P/LPSVs Not Associated with Childhood Malignancy Predisposition or Unmatched Patient Phenotypes

#	Diagnosis	Gene	Variant	P/LPa Increased Risk	Genotype
1	Glioblastoma multiforme	ABCG8	c.55G>C (p.Asp19His)	Increased Risk Allele	Heterozygous
2		CFTR	c.1521_1523del (p.Phe508del)	P	Heterozygous
3	Low-grade glioma	ABCG8	c.55G>C (p.Asp19His)	Increased Risk Allele	Heterozygous
4	Retinoblastoma	ABCG8	c.55G>C (p.Asp19His)	Increased Risk Allele	Heterozygous
5		ATM	c.7699_7702del (p.Asn2567Glufs*5)	P	Heterozygous
6	Retinoblastoma	CFTR	c.1521_1523del (p.Phe508del)	P	Heterozygous
7	Synovial sarcoma	CFTR	c.416A>T (p.His139Leu)	P	Heterozygous
8	Anaplastic large cell lymphoma	CFTR	c.1210-34TG[12]T[5] (Intronic)	P	Heterozygous
9	Osteosarcoma	G6PD	c.563C>T (p.Ser188Phe)	P	Homozygous
10	Mixed cell non germinoma	G6PD	c.563C>T (p.Ser188Phe)	P	Hemizygous
11	Low-grade glioma	GSS	c.4del (p.Ala2Profs*14)	P	Heterozygous
12	Desmoplastic fibroma	CHEK2	c.1100del (p.Thr367Metfs*15)	P	Heterozygous
13		RBM8A	c.67+32G>C (Intronic)	P (low penetrance)	Heterozygous
14	Ewing sarcoma	RBM8A	c.-21G>A (Non-coding)	P (low penetrance)	Heterozygous
15	Retinoblastoma	RBM8A	c.-21G>A (Non-coding)	P (low penetrance)	Heterozygous
16	Ovarian Mature Cystic Teratoma	LZTR1	c.993+1G>T (Splice donor)	LP	Heterozygous
17		RBM8A	c.-21G>A (Non-coding)	P (low penetrance)	Heterozygous
18	Low-grade glioma	SLC2A1	c.653G>A (p.Arg218His)	LP	Heterozygous
19	Ganglioneuroblastoma	SPINK1	c.101A>G (p.Asn34Ser)	Increased Risk Allele	Heterozygous
20	AML	TCN2	c.26del (p.Phe9Serfs*23)	P	Heterozygous
21	Juvenile granulosa cell tumor	ABCG8	c.55G>C (p.Asp19His)	Increased Risk Allele	Heterozygous
22		RBM8A	c.-21G>A (Non-coding)	P (low penetrance)	Heterozygous

23	Low-grade glioma	TNFRSF 13B	c.310T>C (p.Cys104Arg)	LP	Heterozyg ous
24	Low-grade glioma	RBM8A	c.-21G>A (Non-coding)	P (low penetrance)	Heterozyg ous
25	BCP-ALL	TNFRSF 13B	c.204dup (p.Leu69Thrfs*12)	P	Heterozyg ous
26	Low-grade glioma	RECQL4	c.2464-1G>C (Splice acceptor)	P	Heterozyg ous
27	Medulloblastoma	ERCC6L 2	c.2623dup (p.Tyr875Leufs*2)	P	Heterozyg ous
28	Retinoblastoma	CHEK2	c.470T>C (p.Ile157Thr)	P (low penetrance)	Heterozyg ous
29	Low-grade glioma	ATM	c.1065+1G>T (Splice donor)	LP	Heterozyg ous

*Merged rows in the table – the same patient with multiple variants.

Supplementary Table 9- The rates of pediatric cancers in Israel by cancer types

**Cancer Type**	**Estimated % of Total Cases**	**Estimated Annual Cases (Range)**	**Comments**
Leukemia	30%	90–120	Most common pediatric cancer
Brain & CNS Tumors	15–20%	45–80	Includes astrocytoma, medulloblastoma
Lymphomas	10–15%	30–60	Hodgkin and non-Hodgkin
Neuroblastoma	5–10%	15–40	Most often in children <5 years
Wilms Tumor (Kidney)	5–7%	15–28	Usually diagnosed before age 5
Bone Cancers	7–8%	21–32	Osteosarcoma, Ewing’s sarcoma
Soft Tissue Sarcomas	5–6%	15–24	Rhabdomyosarcoma common in younger children
Liver Cancers	3–4%	9–16	Hepatoblastoma more frequent in infants
Germ Cell Tumors	~4%	12–16	Includes testicular and ovarian germ cell tumors
Retinoblastoma	1–2%	3–8	Primarily affects children under age 5
Total (All Cancers)	100%	300–400	Based on national registry estimates
